# Sheep as Host Species for Zoonotic *Babesia venatorum*, United Kingdom

**DOI:** 10.3201/eid2512.190459

**Published:** 2019-12

**Authors:** Alexander Gray, Paul Capewell, Colin Loney, Frank Katzer, Brian R. Shiels, William Weir

**Affiliations:** University of Glasgow, Glasgow, Scotland, UK (A. Gray, P. Capewell, C. Loney, B.R. Shiels, W. Weir);; Moredun Research Institute, Penicuik, Scotland, UK (F. Katzer)

**Keywords:** zoonoses, babesia, babesiosis, tickborne diseases, sheep, cattle, deer, United Kingdom, Scotland, parasites, Babesia venatorum, vectorborne infections

## Abstract

*Babesia venatorum* is an increasingly prominent zoonotic parasite that predominantly infects wild deer. Our molecular examination of *Babesia* infecting mammals in the United Kingdom identified 18S sequences in domestic sheep isolates identical to zoonotic *B. venatorum*. Identification of this parasite in livestock raises concerns for public health and farming policy in Europe.

Babesiosis, an economically consequential animal disease caused by a wide range of tick-transmitted *Babesia* spp. parasites, is recognized as an emerging infection in humans (*1*). *Babesia venatorum* (formerly *Babesia* sp. EU1) is notable in that it appears able to infect humans without immune suppression or splenectomy and can present with more severe symptoms (*2*,*3*). However, the higher prevalence in healthy persons may be a consequence of intensive active sampling; understanding the true impact on healthy patients requires further investigation. Nevertheless, the parasite is increasingly reported in Europe, with 3 confirmed human infections (*3*,*4*). Babesiosis is treatable in most cases (*5*), although successful treatment depends on rapid and accurate diagnosis of the correct *Babesia* species. Diagnosis may be complicated by possible serologic cross-reactivity in laboratory diagnostic tests; *B. venatorum* infections may have been overlooked or misdiagnosed (*1*). Recent serologic reexamination of human babesiosis cases using *B. venatorum* antigen derived from a cloned isolate found that *Babesia* isolates from Europe could be typed without cross-reactivity, indicating the potential for more refined tests (*6*).

Despite identification across continental Europe, *B. venatorum* infecting vertebrate hosts has not been reported in the United Kingdom. Six 18S small subunit (SSU) rRNA sequences with high similarity to *B. venatorum* (99%) have been amplified from ticks infesting dogs and cats in the United Kingdom, but no infected mammalian hosts have been detected despite intensive sampling (*7*,*8*). In continental Europe, roe deer are believed to be the primary vertebrate host (*9*,*10*), although it is possible that livestock could represent a source of infection, as *B. venatorum* has been detected in ticks collected from sheep in Switzerland (*11*) and cattle in Belgium (*12*). Even so, *B. venatorum* infecting these hosts has not been reported, and livestock are not considered a major factor in disease epidemiology.

## The Study

To confirm that *B. venatorum* is present in the United Kingdom and to identify putative vertebrate hosts, we collected blood from sheep (n = 93) and cattle (n = 107) at 2 farms in northeastern Scotland (Appendix Figure), selected because of previous reports of tickborne disease, including red water in cattle (*Babesia divergens* infection) and tickborne fever (*Anaplasma phagocytophilum* infection) and louping ill (ovine encephalomyelitis) in sheep. We also collected blood postmortem from culled wild red deer at site A (n = 24) and 6 surrounding areas (n = 60; Appendix Figure). To provide temporal information, we sampled 34 sheep at site B in both June and November 2014 and sampled 12 sheep in either June or November. The study was approved by the Ethics and Welfare Committee of the University of Glasgow School of Veterinary Medicine (Ref. 15a/13). 

We prepared smears from sheep blood and stained them with May-Grünwald Giemsa stain. We extracted DNA using a Wizard Genomic DNA Purification Kit (Promega, https://www.promega.com) with prior homogenization and incubation with proteinase K (Invitrogen, https://www.thermofisher.com). We amplified the informative hypervariable V4 region of the 18S SSU rRNA gene using nested PCR and previously validated primers/conditions (outer: BT1-F 5′-GGTTGATCCTGCCAGTAGT and BTH-1R 5′-TTGCGACCATACTCCCCCCA [*13*]; inner: RLB-F2 5′-GACACAGGGAGGTAGTGACAAG and RLB-R2 5′-CTAAGAATTTCACCTCTGACAGT [*14*]). We separated amplicons of the predicted size using agarose gel electrophoresis, purified them using a QIAquick PCR purification kit (QIAGEN, https://www.qiagen.com), and had them sequenced commercially (Eurofins Genomics*,* https://www.eurofinsgenomics.com). We deposited the sequences into GenBank (accession ns. MK641004–18) and compared them with the National Center for Biotechnology Information nonredundant database using BLAST (https://blast.ncbi.nlm.nih.gov). We aligned high-scoring hits and constructed a neighbor-joining tree using ClustalW (https://www.genome.jp/tools-bin/clustalw). We assessed tree stability with 1,000 bootstrapping replicates and visualized it using FigTree version 1.4.2 (https://github.com/rambaut/figtree/releases). We included the bovine 18S SSU rRNA sequence as a root.

**Figure 1:**
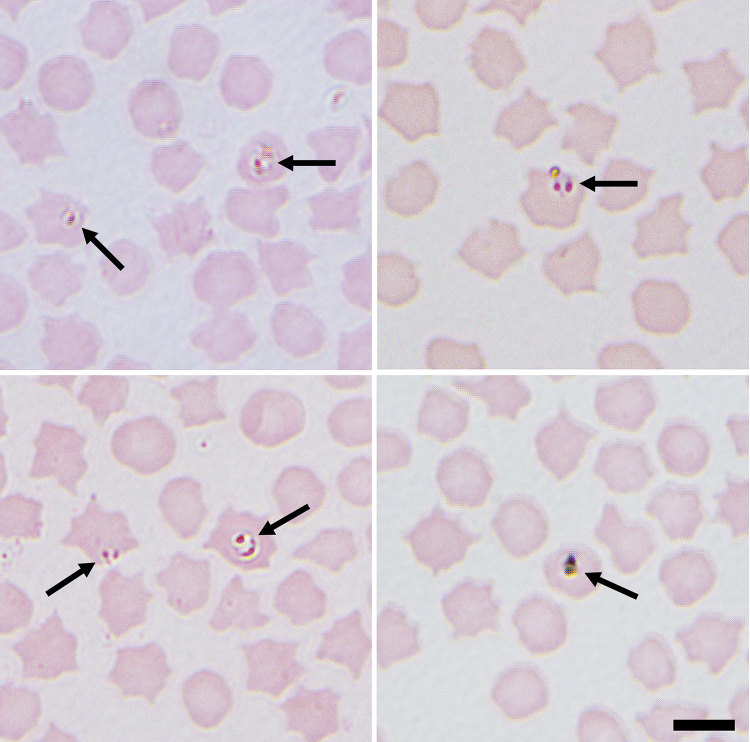
Representative images of small intracellular *Babesia* (arrows) identified in sheep erythrocytes from several sites in northeastern Scotland, UK. Both paired proforms and ring forms are visible. Images were taken at ×1,000 magnification with oil immersion. Scale bar indicates 5 μm.

**Figure 2:**
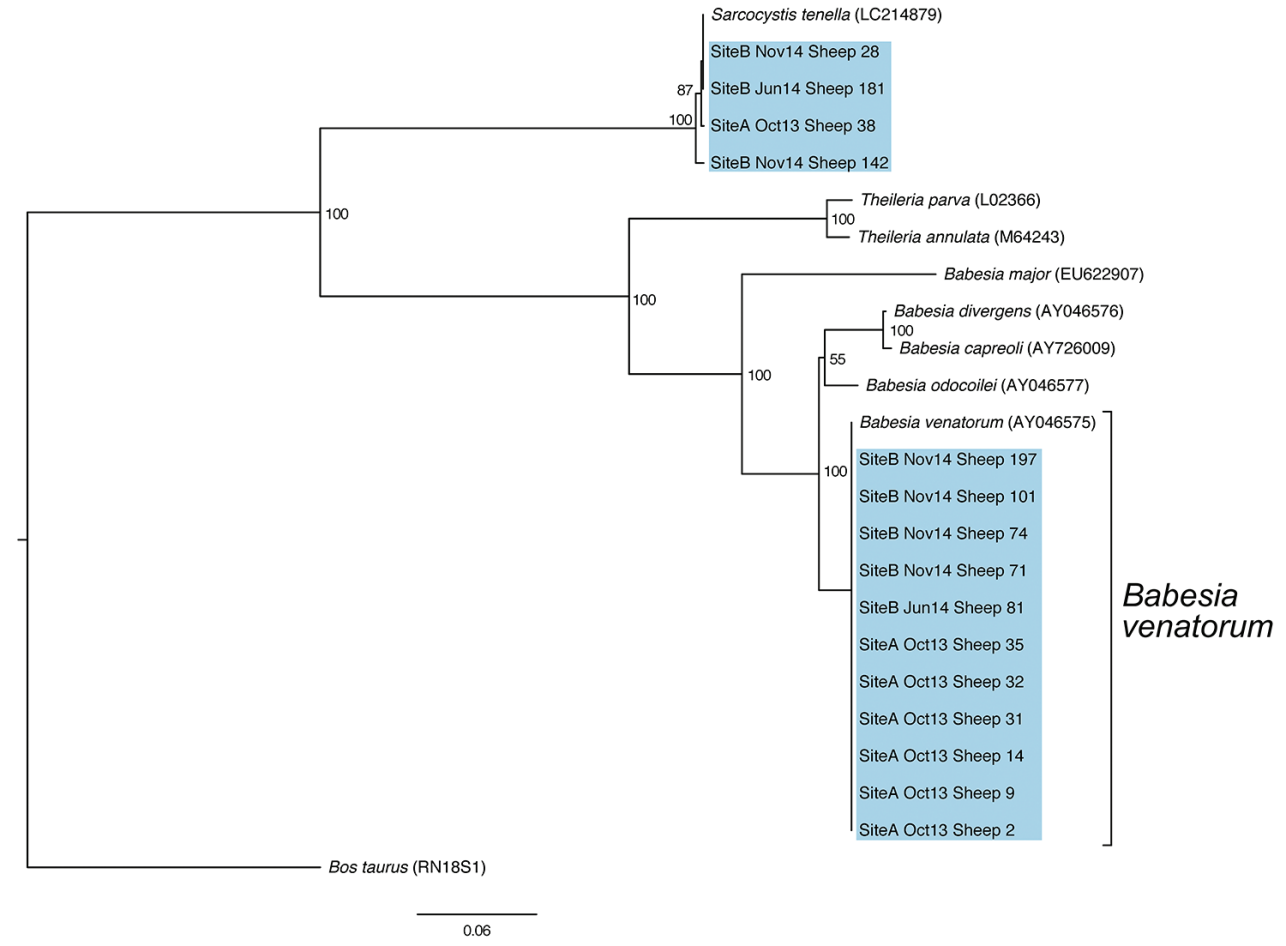
A neighbor-joining tree of 18S small subunit rRNA amplicon sequences obtained from sheep at sites A and B in northeastern Scotland, UK. Blue shading indicates sequences obtained in this study. Previously published *Babesia* and *Theileria* sequences include *B. venatorum* (GenBank accession no. AY046575), *B. divergens* (AY046576), *B. capreoli* (AY726009), *B. odocoilei* (AY046577), *B. major* (EU622907), *Theileria parva* (L02366), and *T. annulata* (M64243). In addition, a *Sarcocystis tenella* (LC214879) isolate was included because of the presence of a similar parasite identified in the sheep population. A *Bos taurus* 18S small subunit rRNA sequence (RN18S1) was used to root the tree.

Initial blood smears from sheep revealed the presence of a small *Babesia* species displaying ring and pyriform morphology in 3 samples (Figure 1). Further PCR and sequencing revealed 11 positive samples that were identical to 30 *Babesia* 18S SSU rRNA sequences in the National Center for Biotechnology Information database annotated as *Babesia* sp. EU1 or *B. venatorum* (Figure 2). These sequences had been amplified from human patients in Austria, Italy, and China. These data demonstrate that *B. venatorum* is indeed present in the United Kingdom and that domestic sheep are a host. 

At the site sampled on 2 separate occasions, most (85%) ewes were negative in June and November 2014 and none were positive at both times. Four (12%) were negative in June and became positive in November, whereas 1 was positive on first sampling but negative on the second. These findings indicate that *B. venatorum* infection is persistent but dynamic within the sheep population; new animals are infected over time, and previously infected animals become PCR negative. 

A separate group of 4 sequences distinct from *B. venatorum* was also obtained in the study and showed high identity to the sheep apicomplexan parasite *Sarcocystis tenella* (Figure 2). No *B. venatorum* infections were detected in the cattle population, despite sharing pasture with infected sheep, and no *B. venatorum* infections were detected in any of the culled red deer.

## Conclusions

This study confirms that *B. venatorum* is present in the United Kingdom, but it remains unclear how the parasite entered the country, because there was no history of imported animals at either farm surveyed. However, the survey sites are situated near the main landing areas for migratory birds coming to the United Kingdom from continental Europe, particularly Norway, and *B. venatorum* has been found in ticks collected from the environment and in migratory birds in Scandinavia (*15*). We postulate that birds could act as an import vector for ticks carrying *B. venatorum*. 

The presence of *B. venatorum* in the United Kingdom represents a new risk to humans working, living, or hiking in areas harboring infected ticks and livestock, particularly sheep. As such, local health and veterinary professionals will need to be aware of the disease if the risk for tickborne disease in the United Kingdom is to be fully understood. Current UK medical inclusion criteria for babesiosis focus on identifying cattleborne *B. divergens*. Going forward, consideration of *B. venatorum,* through careful morphologic description of blood smears and sequencing of informative regions of the 18S SSU rRNA gene, will be necessary for accurate diagnosis and correctly targeted treatment regimens.

Our study has revealed that sheep are a natural host for *B. venatorum* in the United Kingdom. Previously, roe deer were believed to be the main vertebrate host for this parasite in Europe (*9*,*10*). It is unclear why *B. venatorum* has not previously been detected in sheep, although it may be that infection in this host species occurs only in particular foci or is limited to the United Kingdom. Thus, ongoing active surveillance of *Babesia* species in UK livestock would be useful to fully understand the prevalence and transmission of the disease. Such information may be critical for controlling the spread of babesiosis, because sheep are routinely transported large distances (including across international borders) and are closely associated with tick habitats. Our study also suggests that the role that livestock play in *B. venatorum* transmission in continental Europe should be reassessed.

In summary, we have demonstrated that *B. venatorum* is present in the UK sheep population. This finding represents a novel potential threat to animal and human health and demonstrates that livestock may act as a major host for *B. venatorum*, affecting the spread of babesiosis across Europe.

Appendix Map of Scotland indicating the approximate location of sites used in this study for sampling livestock and deer.

## References

[1] Hildebrandt A, Gray JS, Hunfeld K-P. Human babesiosis in Europe: what clinicians need to know. Infection. 2013;41:1057–72. 10.1007/s15010-013-0526-8 24104943

[2] Jiang J-F, Zheng Y-C, Jiang R-R, Li H, Huo Q-B, Jiang B-G, et al. Epidemiological, clinical, and laboratory characteristics of 48 cases of “*Babesia venatorum*” infection in China: a descriptive study. Lancet Infect Dis. 2015;15:196–203. 10.1016/S1473-3099(14)71046-1 25539588

[3] Herwaldt BL, Cacciò S, Gherlinzoni F, Aspöck H, Slemenda SB, Piccaluga P, et al. Molecular characterization of a non-*Babesia divergens* organism causing zoonotic babesiosis in Europe. Emerg Infect Dis. 2003;9:942–8. 10.3201/eid0908.020748 12967491 PMC3020600

[4] Häselbarth K, Tenter AM, Brade V, Krieger G, Hunfeld KP. First case of human babesiosis in Germany - Clinical presentation and molecular characterisation of the pathogen. Int J Med Microbiol. 2007;297:197–204. 10.1016/j.ijmm.2007.01.002 17350888

[5] Babesiosis: Clinical manifestations and diagnosis—UpToDate; 2019 [cited 2019 Mar 6]. https://www.uptodate.com/contents/babesiosis-clinical-manifestations-and-diagnosis

[6] Lempereur L, Shiels B, Heyman P, Moreau E, Saegerman C, Losson B, et al. A retrospective serological survey on human babesiosis in Belgium. Clin Microbiol Infect. 2015;21:96.e1–7. 10.1016/j.cmi.2014.07.004 25636942

[7] Smith FD, Wall LE. Prevalence of *Babesia* and *Anaplasma* in ticks infesting dogs in Great Britain. Vet Parasitol. 2013;198:18–23. 10.1016/j.vetpar.2013.08.026 24055106

[8] Davies S, Abdullah S, Helps C, Tasker S, Newbury H, Wall R. Prevalence of ticks and tick-borne pathogens: *Babesia* and *Borrelia* species in ticks infesting cats of Great Britain. Vet Parasitol. 2017;244:129–35. 10.1016/j.vetpar.2017.07.033 28917304

[9] Michel AO, Mathis A, Ryser-Degiorgis M-P. *Babesia* spp. in European wild ruminant species: parasite diversity and risk factors for infection. Vet Res (Faisalabad). 2014;45:65. 10.1186/1297-9716-45-65 24925474 PMC4070358

[10] Zanet S, Trisciuoglio A, Bottero E, de Mera IG, Gortazar C, Carpignano MG, et al. Piroplasmosis in wildlife: *Babesia* and *Theileria* affecting free-ranging ungulates and carnivores in the Italian Alps. Parasit Vectors. 2014;7:70. 10.1186/1756-3305-7-70 24533742 PMC3929754

[11] Hilpertshauser H, Deplazes P, Schnyder M, Gern L, Mathis A. *Babesia* spp. identified by PCR in ticks collected from domestic and wild ruminants in southern Switzerland. Appl Environ Microbiol. 2006;72:6503–7. 10.1128/AEM.00823-06 17021198 PMC1610307

[12] Lempereur L, Lebrun M, Cuvelier P, Sépult G, Caron Y, Saegerman C, et al. Longitudinal field study on bovine *Babesia* spp. and *Anaplasma phagocytophilum* infections during a grazing season in Belgium. Parasitol Res. 2012;110:1525–30. 10.1007/s00436-011-2657-0 21947341

[13] Criado-Fornelio A, Martinez-Marcos A, Buling-Saraña A, Barba-Carretero JC. Molecular studies on *Babesia, Theileria* and *Hepatozoon* in southern Europe: part I. Epizootiological aspects. Vet Parasitol. 2003;113:189–201. 10.1016/S0304-4017(03)00078-5 12719133

[14] Georges K, Loria GR, Riili S, Greco A, Caracappa S, Jongejan F, et al. Detection of haemoparasites in cattle by reverse line blot hybridisation with a note on the distribution of ticks in Sicily. Vet Parasitol. 2001;99:273–86. 10.1016/S0304-4017(01)00488-5 11511414

[15] Hasle G, Leinaas HP, Røed KH, Øines Ø. Transport of *Babesia venatorum*-infected *Ixodes ricinus* to Norway by northward migrating passerine birds. Acta Vet Scand. 2011;53:41. 10.1186/1751-0147-53-41 21699719 PMC3132728

