# Supplementary material for: Sheep as Host Species for Zoonotic Babesia venatorum, United Kingdom
Source: Emerg Infect Dis. 2019 Dec;25(12):2257–60. doi: 10.3201/eid2512.190459 (PMC6874260; doi:10.3201/eid2512.190459)
Supplement: Appendix — Map of Scotland indicating the approximate location of sites used in this study for sampling livestock and deer. [file 19-0459-Techapp-s1.pdf]

Article DOI: https://doi.org/10.3201/eid2512.190459

Sheep as Host Species for Zoonotic *Babesia venatorum*, United Kingdom

Appendix

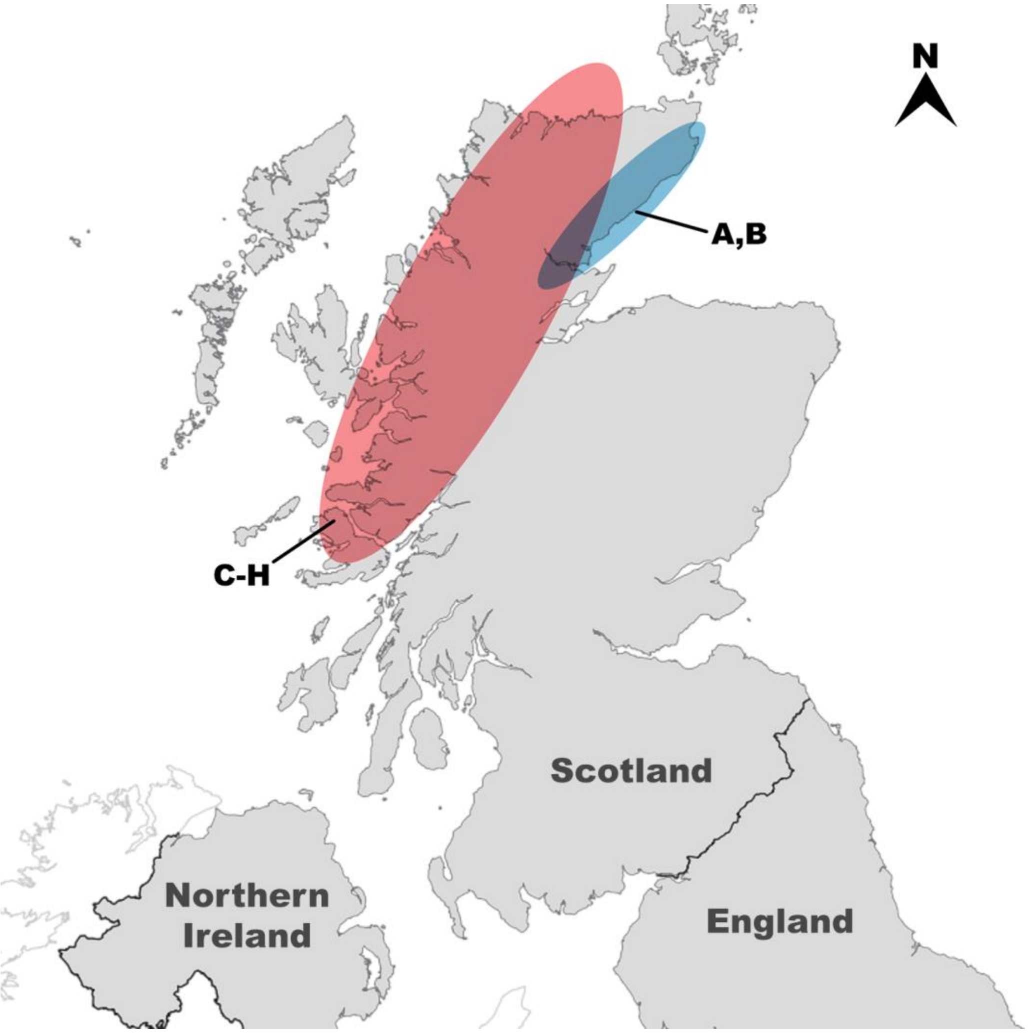

Appendix Figure. Locations of the 8 sampling sites used in this study (A–H). The approximate location of the 2 farm sites (A and B) is marked in blue and the area containing the deer cull sites (C–H) is marked in red.
